# IL-27p28 Production by XCR1^+^ Dendritic Cells and Monocytes Effectively Predicts Adjuvant-Elicited CD8^+^ T Cell Responses

**DOI:** 10.4049/immunohorizons.1700054

**Published:** 2018-01-01

**Authors:** Augustus M. Kilgore, Seth Welsh, Elizabeth E. Cheney, Alisha Chitrakar, Trevor J. Blain, Benjamin J. Kedl, Chris A. Hunter, Nathan D. Pennock, Ross M. Kedl

**Affiliations:** * Department of Immunology and Microbiology, School of Medicine, University of Colorado Denver at Anschutz Medical Campus, Denver, CO 80045; † Department of Pathobiology, School of Veterinary Medicine, University of Pennsylvania, Philadelphia, PA 19104; ‡ Department of Cell, Developmental & Cancer Biology, Oregon Health & Science University, Portland, OR 97239

## Abstract

It is well accepted that the innate response is a necessary prerequisite to the formation of the adaptive response. This is true for T cell responses against infections or adjuvanted subunit vaccination. However, specific innate parameters with predictive value for the magnitude of an adjuvant-elicited T cell response have yet to be identified. We previously reported how T cell responses induced by subunit vaccination were dependent on the cytokine IL-27. These findings were unexpected, given that T cell responses to an infection typically increase in the absence of IL-27. Using a novel IL-27p28–eGFP reporter mouse, we now show that the degree to which an adjuvant induces IL-27p28 production from dendritic cells and monocytes directly predicts the magnitude of the T cell response elicited. To our knowledge, these data are the first to identify a concrete innate correlate of vaccine-elicited cellular immunity, and they have significant practical and mechanistic implications for subunit vaccine biology.

## INTRODUCTION

The last two decades have seen an explosion of information relative to the molecular and cellular functions dictating robust T cell immunity. Mouse models using experimental infectious agents, such as lymphocytic choriomeningitis virus, HSV-1, vaccinia virus, or *Listeria monocytogenes*, have been irreplaceable assets in establishing these mechanistic underpinnings. Further, studies in humans using live attenuated vaccines, such as the yellow fever vaccine, are generally consistent with the responses observed in mouse studies (1). Substantial agreement between mice and humans has also been observed in their responses to vaccination with nonlive adjuvanted subunit vaccines, at least with respect to B cell responses (2).

In contrast, clinically relevant T cell responses to experimental or U.S. Food and Drug Administration–approved subunit vaccines (defined as a nonlive noninfectious formulation consisting of an antigenic target and an adjuvant) have been difficult to generate and/or detect (3). Given the robust cellular responses against infectious challenge, a reasonable assumption is that subunit vaccine formulations will better achieve cellular responses by following the established rules governing the response to infections. That said, concrete correlates of the infectious process to the magnitude of the T cell response have been difficult to clarify, especially for adaptation to subunit vaccine formats. For example, infectious agents often induce IL-12 and/or type I IFN, both of which are “signal 3” cytokines that are known to facilitate maximal CD8^+^ T cell responses to the infection (4–6). However, although it does influence the differentiation process, the amount of IL-12/ type I IFN induced during the infection is not known to correlate with the magnitude of the T cell response. Thus, its predictive value for prioritizing vaccine adjuvants and subunit vaccine formulations capable of eliciting T cell responses is unclear at best. Indeed, there is no clear metric for adjuvant activity that can reasonably predict subsequent cellular immunity, representing a significant challenge to successfully screening for such adjuvants. To address this shortcoming, we present data that IL-27 expression is an effective correlate for adjuvant-elicited cellular immunity.

IL-27 is a pleiotropic cytokine of the IL-12 cytokine family (7, 8). The cytokine itself is composed of an IL-27p28 subunit and the protein EBI3, a subunit shared with IL-35 (9, 10). The IL-27p28/EBI3 heterodimer signals uniquely through a heterodimeric receptor composed of the IL-27Rα and GP130 protein subunits, transducing signals through STAT1, STAT3, and MAPK cascade (11). IL-27Rα is constitutively expressed on multiple cell types, including T cells, B cells, dendritic cells (DCs), and macrophages (12), and is rapidly upregulated after cellular activation. Although there is some evidence that p28 can have functions outside of its dimerization with EBI3 (13, 14), p28 expression is reasonably indicative of IL-27 activity. Although IL-27 has pro-and anti-inflammatory properties (7, 15–19), the global loss of IL-27 in an infectious setting often leads to substantial immune pathology mediated by unchecked CD4 T cell activation and expansion (7, 18, 19). In sharp contrast, we recently identified a key role for IL-27 in facilitating maximal T cell responses to adjuvanted subunit vaccination (20). In the absence of T cell–intrinsic IL-27R expression, the CD4 and CD8 T cell response is heavily compromised to a wide range of vaccine adjuvants. This was unexpected, although consistent with the role of IL-27 in promoting anticancer CD8^+^ T cell responses (21–24).

Modern vaccine adjuvants largely target one or more innate receptors on DCs and other cells of the innate immune response. The T cell responses to each of these innate receptor-based adjuvants is IL-27 dependent (20), although the responses vary considerably. We hypothesized that the variability in magnitude of the T cell responses to these adjuvants might be related to the capacity of each adjuvant to instigate IL-27p28 production. Toward that end, we created a reporter mouse that expresses eGFP under the control of the IL-27p28 promoter. We analyzed eGFP expression in vivo after injection of innate receptor-based vaccine adjuvants and determined its correlation with the magnitude of the subsequent CD8^+^ T cell response. We found that eGFP expression in XCR1^+^ DCs and Ly6C^hi^ monocytes was highly predictive of the ensuing adaptive CD8^+^ T cell response. These results indicate that the capacity to induce IL-27p28 expression should be used in screening for adjuvants capable of eliciting T cell responses.

## MATERIALS AND METHODS

### IL-27p28–eGFP mice

The 8139-bp sequence directly upstream of the ATG start codon of the mouse *il-27p28* gene (Ensembl), containing two previously documented regulatory regions for *il-27p28* expression (25–27), was placed in-frame with the ATG start codon of *eGFP* using the pRED-ET λ phage recombineering approach (catalog number K005; Gene Bridges). The bacterial backbone containing eGFP was obtained from Addgene (pUCBB-eGFP), whereas the bacterial artificial chromosomes (BACs) containing the mouse chromosomal regions of *il-27p28* were obtained from the Children’s Hospital of Oakland Research Institute BACPAC Resource Center. A short modified simian virus long poly-A sequence (5′-AATAAACAAGTTAACAACAACAATTGCATTCATTTTATGTTTCAGGTTCAGGGGGAGGTGTGGGAGTTTTTT-3′) was attached to the 3′ end of the bacterial *eGFP* to provide mammalian mRNA stability (28). The 3′ end of the poly-A sequence was followed by a loxp-neo-loxp insertion (cloned from plasmid PL45.2; Gene Bridges) to allow for postembryonic integration removal of tandem insertions by cre expression. The complete plasmid sequence is available. C57BL/6 (B6) blastocysts were injected with linearized plasmid and implanted into pseudo-pregnant albino B6 females. The resulting chimeric pups were bred to wild-type (WT) B6 mates, and the pups screened for the presence of transgene by PCR using the following primers (5′-CTGACATGTGAGCAAGGGCGA-3′ (IL-27p28 YFP RT probe), 5′-TAGCCAGGGAAGACTTAGTGA-3′ (IL-27p28 YFP RT forward), and 5′-CCGTCCAGCTCGACCAG-3′ (IL-27p28 YFP RT reverse). A founder was identified, and the pups were further crossed to WT B6 mice to obtain transgenic and nontransgenic littermate controls.

### Immunization

For all 6-, 8-, 10- and 12-h eGFP experiments, male and female IL-27p28–eGFP^+^ mice were immunized with 25–100 μg (as indicated) of innate receptor agonist in 200 μl of 1× PBS containing 150 μg of detoxified (29) (LPS-free as determined by limulus assay) whole chicken OVA (Sigma) via i.p. injection. For day-7 tetramer experiments, male and female B6 or IL-27p28–eGFP^−^ littermate (BL/6 background) mice were immunized i.v. or i.p., as previously described (20). Three or four mice were vaccinated for each adjuvant listed. The following doses of adjuvants were used: lipoteichoic acid (LTA; 100 μg; InvivoGen), Pam3Cys (25 μg; InvivoGen), polyinosinic-polycytidylic acid (polyIC; 50 μg; GE), flagellin (8.3 μg; InvivoGen), CpG (50 μg; InvivoGen), MPL (40 μg; InvivoGen), and 3M-012 (50 μg). rIL-27 injections were delivered i.v. (10 μg per mouse; Sino Biological).

### DC isolation, tetramer staining, and flow cytometry

Animals were euthanized at 6, 8, 10, or 12 h postimmunization or on day 7 postimmunization for all subunit vaccinations. Spleens were digested, as previously described (30), using 1 mg/ml Collagenase D (Roche) and 50 μg/ml DNase (Worthington) to generate a single-cell suspension. Intracellular cytokine staining was performed by incubating splenocytes for 6 h with 5 μg/ml Brefeldin A (Enzo Life Sciences), surface staining, fixing with 1% paraformaldehyde, and cytokine staining in 1× Perm Buffer (Invitrogen). Flow cytometry data were obtained using a Cyto-FLEX (Beckman Coulter) flow cytometer, and analysis was performed using FlowJo software (PC version 10.1r7). The following cell surface Abs and clones were used for DC staining: PerCP anti-mouse CD8 (53-6.7; BioLegend), allophycocyanin anti-mouse CD64 (X54-5/7.1; BioLegend), Ghost Dye Red 780 (Tonbo), BV421 anti-mouse CD11c (N418; BioLegend ), BV510 anti-mouse Ly6G (1A8; BioLegend), biotin anti-mouse MHC class II (M5/ 114.15.2; BioLegend), BV605 streptavidin (BioLegend), redFluor 710 anti-mouse B220 (RA3-6B2; Tonbo), Alexa Fluor 700 anti-mouse CD3 (17A2; BioLegend), PE anti-mouse XCR1 (ZET; BioLegend), and PE-Cy7 anti-mouse CD11b (M1/70; Tonbo). The following Abs and clones were used for monocyte and granulocyte staining: PerCP anti-mouse CD11c (N418; BioLegend), allophycocyanin anti-mouse CD64 (X54-5/7.1; BioLegend), Ghost Dye Red 780 (Tonbo), BV421 anti-mouse Ly6C (HK1.4; BioLegend), BV510 anti-mouse Ly6G (1A8; BioLegend), biotin anti-mouse MHC class II (M5/114.15.2; BioLegend), BV605 streptavidin (BioLegend), redFluor 710 anti-mouse B220 (RA3-6B2; Tonbo), Alexa Fluor 700 anti-mouse CD3 (17A2; BioLegend), PE anti-mouse F4/80 (BM8; BioLegend), and PE-Cy7 anti-mouse CD11b (M1/70; Tonbo). The following Abs and clones were used for tetramer staining: allophycocyanin or PE H-2Kb+SIINFEKL (National Institutes of Health Tetramer Core), FITC anti-mouse KLRG1 (2F1/KLRG1; BioLegend), redFluor 710 anti-mouse B220 (RA3-6B2; Tonbo), PE-Cy7 anti-mouse CD3 (17A2; BioLegend), BV421 anti-mouse CD8 (53-6.7; BioLegend), BV711 anti-mouse CD127 (A7R34; BioLegend), Ghost Dye Red 780 (Tonbo), and PerCP anti-mouse CD44 (IM7; BioLegend). The following Abs and clones were used for intracellular cytokine staining (ICCS): BV421 anti-mouse CD8 (53-6.7; BioLegend), Ghost Dye Red 780 (Tonbo), redFluor 710 anti-mouse B220 (RA3-6B2; Tonbo), PE-Cy7 anti-mouse CD11b (M1/70; Tonbo), biotin anti-mouse MHC class II (M5/114.15.2; BioLegend), BV605 streptavidin (BioLegend), PE anti-mouse XCR1 (ZET; BioLegend), FITC anti-mouse CD11c (HL3; BD), BV510 anti-mouse CD3 (17A2; BioLegend), allophycocyanin anti-mouse IL-12 (C15.6; BD), Alexa Fluor 647 anti-mouse IL-27 (MM27-7B1; BioLegend), and PerCP anti-mouse CD86 (GL-1; BioLegend). Graphing of data and assessment for statistical significance were performed using Prism (Graph-Pad Software, La Jolla, CA).

### Fluorescent microscopy

Spleens from immunized and control IL-27p28–eGFP transgenic mice were removed 6 h after polyIC administration and immediately fixed overnight using PLP buffer, as previously described (30). Spleens were flash frozen with liquid nitrogen in OCT and 20-μm tissue sections were obtained using a cryostat. Sections were left to dry for 30 min, blocked with 5% BSA for 1 h, and stained with anti-eGFP (Alexa Fluor 488 Rabbit polyclonal; Life Technologies), XCR1 (allophycocyanin clone ZET; BioLegend), and B220 (PE RA3-6B2; BioLegend) in 5% BSA. After washing extensively, sections were coverslipped with Fluoromount mounting medium and left to dry overnight. Slides were imaged on a Zeiss LSM 780 confocal microscope, and image analysis was performed with Fiji (31).

### Quantitative real-time PCR

Animals were euthanized at 6, 8, 10, or 12 h postimmunization, and collagenase digestion of spleens was performed as described above. DCs were enriched by positive selection using allophycocyanin anti-mouse CD11c (BioLegend) and anti-allophycocyanin MicroBeads, according to the manufacturer’s instructions (Miltenyi Biotec). Cells were stained will the following Abs: PE anti-mouse XCR1 (BioLegend), allophycocyanin anti-mouse CD11c (BioLegend), BV421 anti-mouse B220 (BioLegend), BV421 anti-mouse CD3 (BioLegend), and BV421 SYTOX Blue (Thermo Fisher). Cells were then sorted on a BD FACSAria into “eGFP low” and “eGFP high” populations after selection for live, CD11c/XCR1^+^, B220/CD3^−^ cells. RNA was prepared from the sorted cells using an RNeasy Plus Mini Kit, according to manufacturer instructions (QIAGEN). cDNA was prepared from these samples using an Invitrogen SuperScript III First-Strand Synthesis System, according to the manufacturer’s instructions (Thermo Fisher). Quantitative PCR was conducted on these samples using a LightCycler 480 (Roche), according to the following program: 95°C for 10 min and 40 cycles of 95°C for 15 s, 54°C for 20 s, and 68°C for 20 s, with 4°C hold. The following primers were used: 5′-ACGTAAACGGCCACAAGTTC-3′(eGFPforward),5′-AAGTCGTGCTGCTTCATGTG-3′ (eGFP reverse), 5′-AACTTTGGCATTGTGGAAGG-3′ (GAPDH forward), 5′-ACACATTGGGGGTAGGAACA-3′ (GAPDH reverse), 5′-CTCTGCTTCCTCGCTACCAC-3′ (p28 forward), and 5′-GGGGCAGCTTCTTTTCTTCT-3′ (p28 reverse). Data were analyzed according to the 2^ΔΔCT^ method (32).

## RESULTS

### IL-27p28–eGFP mouse effectively reports IL-27 expression

**FIGURE 1:**
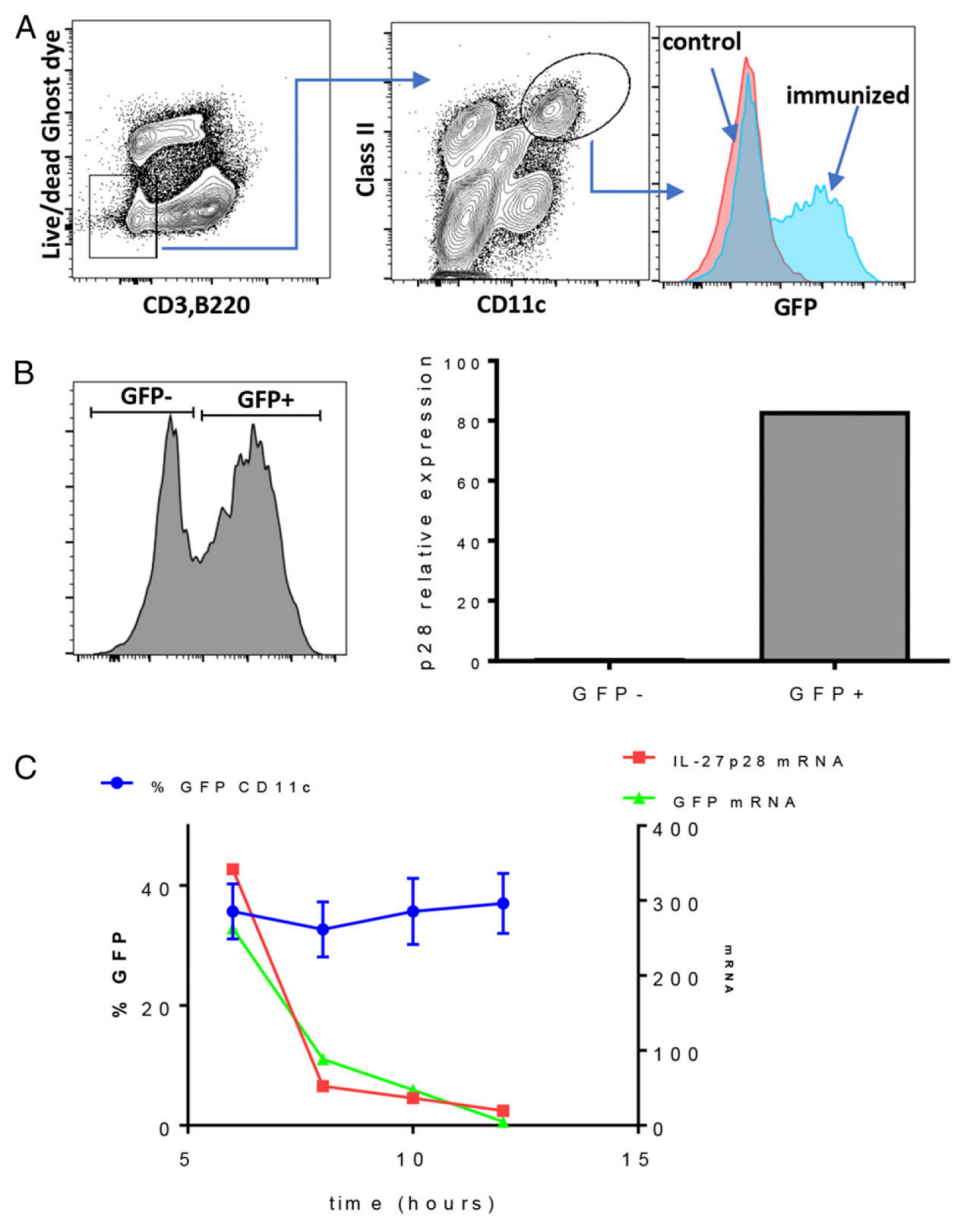
eGFP expression faithfully reports IL-27p28 transcription (**A**) Mice were immunized i.v. with combined polyIC/anti-CD40, and 6 h later the spleens were harvested, digested with collagenase, and stained with Abs to identify eGFP expression in conventional DCs. (**B**) Cells from mice immunized as in (A) were flow sorted for eGFP^+^ and eGFP^−^ DCs using the markers as shown. RNA was isolated from the sorted cells, and quantitative real-time PCR (qRT-PCR) for IL-27p28 was performed as described in *Materials and Methods*. The experiment was done twice, with qRT-PCR performed on cells sorted and pooled from three mice. (**C**) Spleen cells isolated at the indicated time points from mice immunized as in (A) were analyzed for the percentage eGFP^+^ conventional DCs gated as in (A) (blue symbols, left *y*-axis). At each time point, eGFP^+^ cells were flow sorted, RNA was isolated, and qRT-PCR for IL-27p28 (red symbols, right *y*-axis) and eGFP (green symbols, right *y*-axis) message was performed. This experiment was performed twice, error bars for the percentage of eGFP^+^ DCs indicate SD derived from three mice per time point. qRT-PCR for IL-27p28 and eGFP at each time point was again performed on cells sorted and pooled from three mice.

Given the importance of IL-27 in vaccine-elicited cellular responses (20), we made a transgenic reporter host expressing eGFP under the control of the IL-27p28 promoter. Using λ phage recombination techniques, we extracted an 8.1-kb region of the 5′ promoter of the IL-27p28 gene, known to contain regulatory elements for IL-27p28 expression, from the appropriate mouse BAC and place it in-frame upstream of the eGFP gene in an eGFP backbone plasmid (Supplemental Fig. 1A). Following traditional transgenic techniques, the construct was linearized and inserted into blastocytes through microinjection. Pups from one of the two founders identified were screened for the BAC transgene by genomic PCR. Initially, we sought to validate the transgene for induction of eGFP and effective reporting of IL-27p28 expression. Mice were immunized with a combination adjuvant that induces potent CD4^+^ and CD8^+^ T cell expansion (polyIC + agonistic anti-CD40 Ab) (20, 33, 34), and eGFP expression in the spleen was analyzed 6 h later using flow cytometry. After collagenase/DNase digestion to release activated DCs and monocytes, we identified eGFP-expressing DCs by gating on CD3/B220^−^ class II^+^CD11c^+^ cells (Fig. 1A). To confirm that eGFP was faithfully reporting IL-27p28 expression, we again immunized mice with polyIC/anti-CD40; at 6 h, we used the same gating strategy to identify DCs and then sorted for eGFP^+^ and eGFP^−^ cells (Fig. 1B). Quantifying the *il-27p28* message in each sorted population by quantitative real-time PCR (qRT-PCR) revealed *il-27p28* expression in eGFP^+^ DCs but not in eGFP^−^ DCs (Fig. 1B). This indicated that eGFP expression was easily detectable within DCs after vaccine adjuvant administration and that it was effectively reporting the expression of IL-27p28.

Although IL-27p28 expression clearly resided within the eGFP-expressing cells, we did not yet have an understanding of the time frame over which eGFP reflected actual IL-27p28 message. Because we had not used destabilized *GFP* for making the IL-27p28 reporter host, we expected that the presence of eGFP might last longer in activated DCs than the message for IL-27p28. Therefore, we examined eGFP expression within total DCs as gated above (CD3/B220^−^ , class II^hi^, CD11c^+^) to determine the overall time course and concordance in expression for eGFP and IL-27p28. Spleens were harvested from the mice at 6, 8, 10, and 12 h after polyIC/anti-CD40 administration, and DCs were analyzed by flow cytometry to identify the percentage expressing eGFP and the mean fluorescence intensity of eGFP expression (Fig. 1B). Additionally, eGFP^+^ and eGFP^−^ DCs were flow sorted, the RNA was recovered, and qRT-PCR was performed to quantify *il-27p28* and eGFP mRNA within the eGFP^+^ fraction (Fig. 1C). eGFP protein expression was detectable as early as 4 h (data not shown), reached maximal expression by 6–8 h after immunization (Fig. 1C), and remained relatively stable between 12 and 24 h (data not shown). In contrast, *il-27p28* and eGFP message peaked at 6 h and declined sharply and rapidly thereafter (Fig. 1C); it was barely detectable 12 h after immunization. These results indicated that eGFP faithfully identifies the cells expressing IL-27p28 after adjuvant administration, but that eGFP fluorescence is more stable than *il-27p28* transcript levels. Collectively, we conclude that eGFP fluorescence accurately identifies cells actively transcribing *il-27p28* for 6–12 h after adjuvant administration, after which it becomes a useful marker for the cells that previously had made IL-27p28. It is worth noting that ICCS for IL-27p28 in DCs at 6 and 24 h after immunization was consistent with *il-27p28* transcripts in WT B6 and IL-27p28–eGFP mice (Supplemental Fig. 1B). In addition to demonstrating the greater sensitivity of the IL-27p28–eGFP reporter for monitoring IL-27p28 expression, these data confirm that that the reporter host does not show aberrant production of IL-27p28 relative to a WT host.

### IL-27p28 is primarily expressed in XCR1^+^ DCs and monocytes after adjuvant administration

**FIGURE 2:**
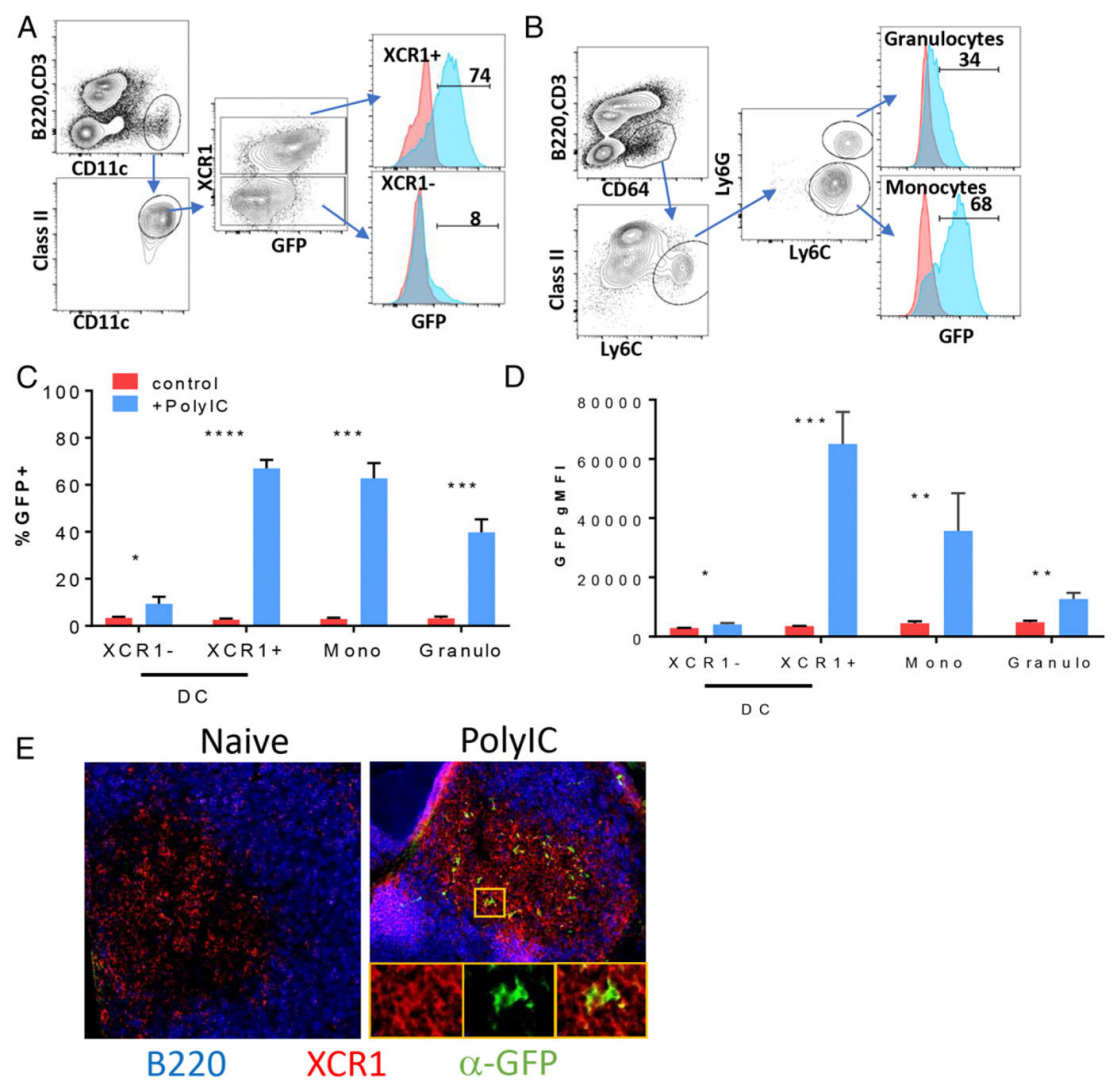
IL-27–eGFP is highly expressed by cDC1 cells and Ly6C^hi^ monocytes after vaccine adjuvant administration Mice were immunized i.p. with combined polyIC/anti-CD40, and 6 h later spleens were harvested, digested in collagenase, and stained with Abs targeting markers for conventional DCs, monocytes, and granulocytes. (**A** and **B**) Using the gating strategies shown, eGFP expression was compared between unimmunized (red line graphs) and immunized (blue line graphs) reporter mice for cDC1 and cDC2 cells (A) or monocytes and granulocytes (B). The line graph marker was set based on the unimmunized sample, and the number represents the percentage of eGFP^+^ cells in the immunized sample. Quantification of the percentage of eGFP^+^ cells in each subset (**C**) and gMFI of eGFP in each subset (**D**). Error bars indicate SD derived from three mice each, in controls and immunized. (**E**) Spleens from mice immunized as in (A) were removed and fixed, and frozen sections were stained as described in *Materials and Methods* for B220 (blue), XCR1 (red), and eGFP (green). Original magnification ×22. Insets show magnified area marked by the yellow box. The experiment was performed three or four times, depending on the adjuvant. *p < 0.05, **p < 0.01, ***p < 0.001, ****p < 0.0001, *t* test.

We next determined which hematopoietic cells expressed IL-27/eGFP after challenge with different adjuvants. To initially establish what cell types were producing eGFP, mice were immunized with polyIC, and splenocytes were analyzed 6 h later by flow cytometry for eGFP in various lineages. Total splenic T and B cells showed essentially no expression of eGFP above unimmunized controls (Supplemental Fig. 2A). In contrast, DCs (Fig. 2A) and Ly6C^hi^ monocytes (Fig. 2B) showed significant expression of eGFP. The two major DC subsets in the spleen, cDC1 and cDC2 cells (35), are both CD11c^+^, class II^hi^, but they express CD8^+^ or CD11b^+^, respectively. cDC1 cells differ from cDC2 cells with regard to their expression of the chemokine receptor XCR1 (36, 37), so we used this marker to differentiate the two subsets (Fig. 2A). Only ~3–5% of cDC2 cells (XCR1^−^) induced eGFP expression after polyIC immunization (Fig. 2A, 2C). In contrast, 60–80% of cDC1 cells (XCR1^+^) were eGFP^+^, as were Ly6c^hi^ monocytes (Fig. 2A, 2C). However, cDC1 cells expressed the most eGFP, as measured by eGFP geometric mean fluorescence intensity (gMFI) (Fig. 2D). Sorting on various levels of eGFP revealed that eGFP gMFI correlated with the relative amount of IL-27 message (Supplemental Fig. 2B), indicating that cDC1 cells express the highest amount of IL-27p28 on a per-cell basis. This conclusion was additionally supported by immunofluorescence analysis of frozen spleen sections, showing high levels of eGFP in XCR1^+^ cells within the T cell zone of the white pulp (Fig. 2E). Interestingly, Ly6G^hi^ granulocytes also showed eGFP expression (Fig. 2B–D), but this depended on which adjuvant was administered (see below).

**FIGURE 3:**
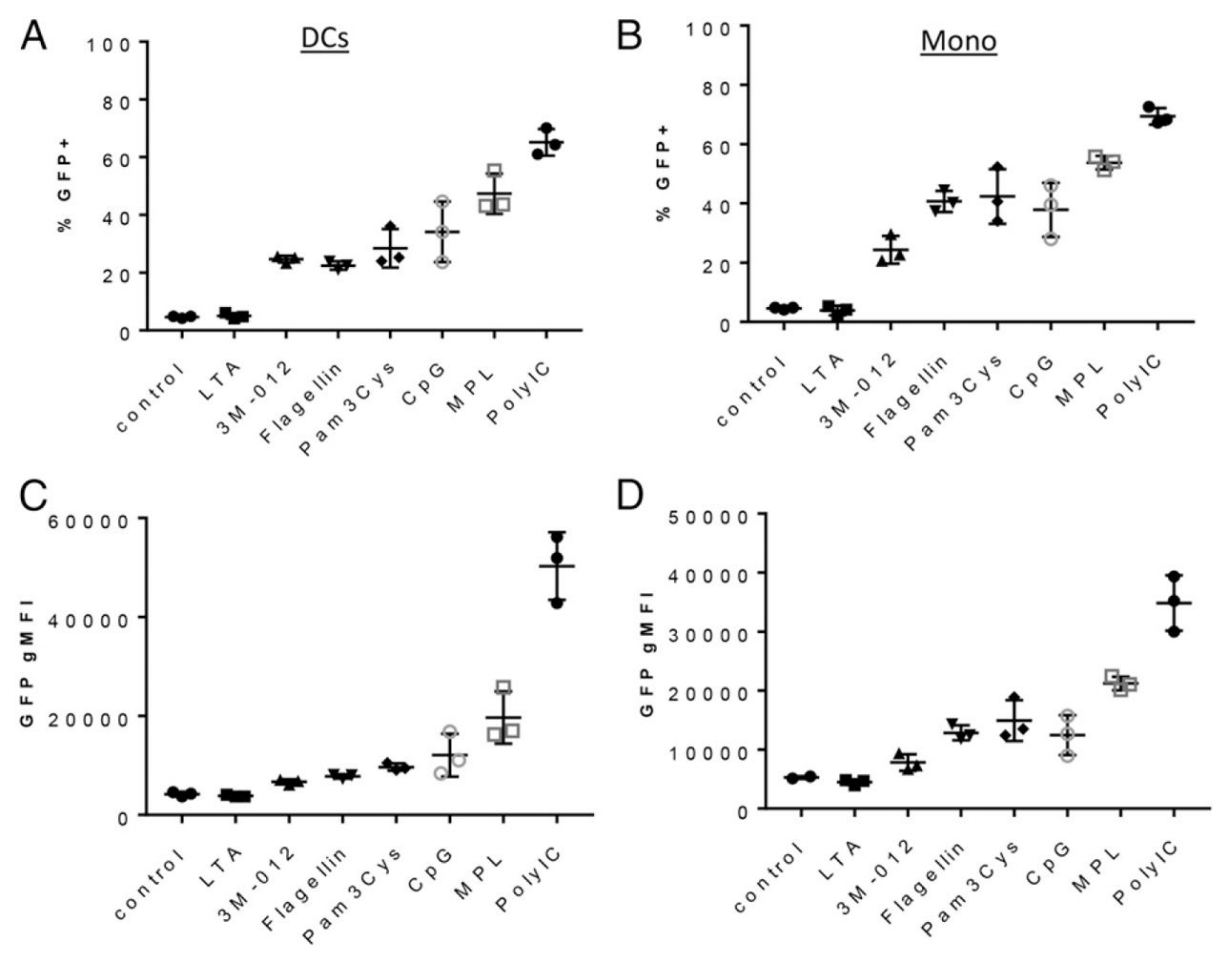
Adjuvants targeting innate receptors induce a broad range of IL-27–eGFP Mice were immunized i.p. with the indicated adjuvants, and the spleens were harvested 6 h later. Splenocytes were isolated by collagenase digestion and stained to identify cDC1 cells (**A** and **C**) and monocytes (**B** and **D**), as described in Fig. 2. DCs and monocytes were evaluated for the percentage of eGFP^+^ cells (A and B) and eGFP gMFI (C and D). The experiment was performed twice; error bars indicate SD derived from three mice each.

With the dominant populations of eGFP-expressing cells established, we next examined the potency of different adjuvants to induce IL-27/eGFP in cDC1 cells and monocytes. We challenged IL-27p28–eGFP mice with a series of adjuvants commonly used in experimental settings, and 6 h later assessed eGFP expression in DCs and monocytes, as before (Fig. 3). The range of IL-27p28–eGFP induction varied considerably among the adjuvants, from LTA inducing eGFP at levels barely detectable over no adjuvant controls, to polyIC often inducing >80% eGFP^+^ cDC1 cells (Fig. 3A, 3C) and monocytes (Fig. 3B, 3D). In addition, there was a strong correlation between the percentage of eGFP expression and the overall gMFI of cDC1 cells or monocytes. Interestingly, neutrophils/ granulocytes could be induced to express eGFP, but only by polyIC and not by any of the other adjuvants administered (Supplemental Fig. 3A). The ability of polyIC to induce IL-27p28 from granulocytes is unlikely to be solely a matter of type I IFN induction, because TLR7 agonists also induce a potent type I IFN response, but minimal IL-27/eGFP, in granulocytes (Supplemental Fig. 3A). We concluded from these data that cDC1 cells and inflammatory monocytes are consistently the main cell types to express IL-27/eGFP after adjuvant administration and that the level of IL-27/eGFP induction varies considerably over a broad range of adjuvants.

### IL-27/eGFP expression predicts the magnitude of adjuvant-elicited CD8^+^ T cell responses

**FIGURE 4:**
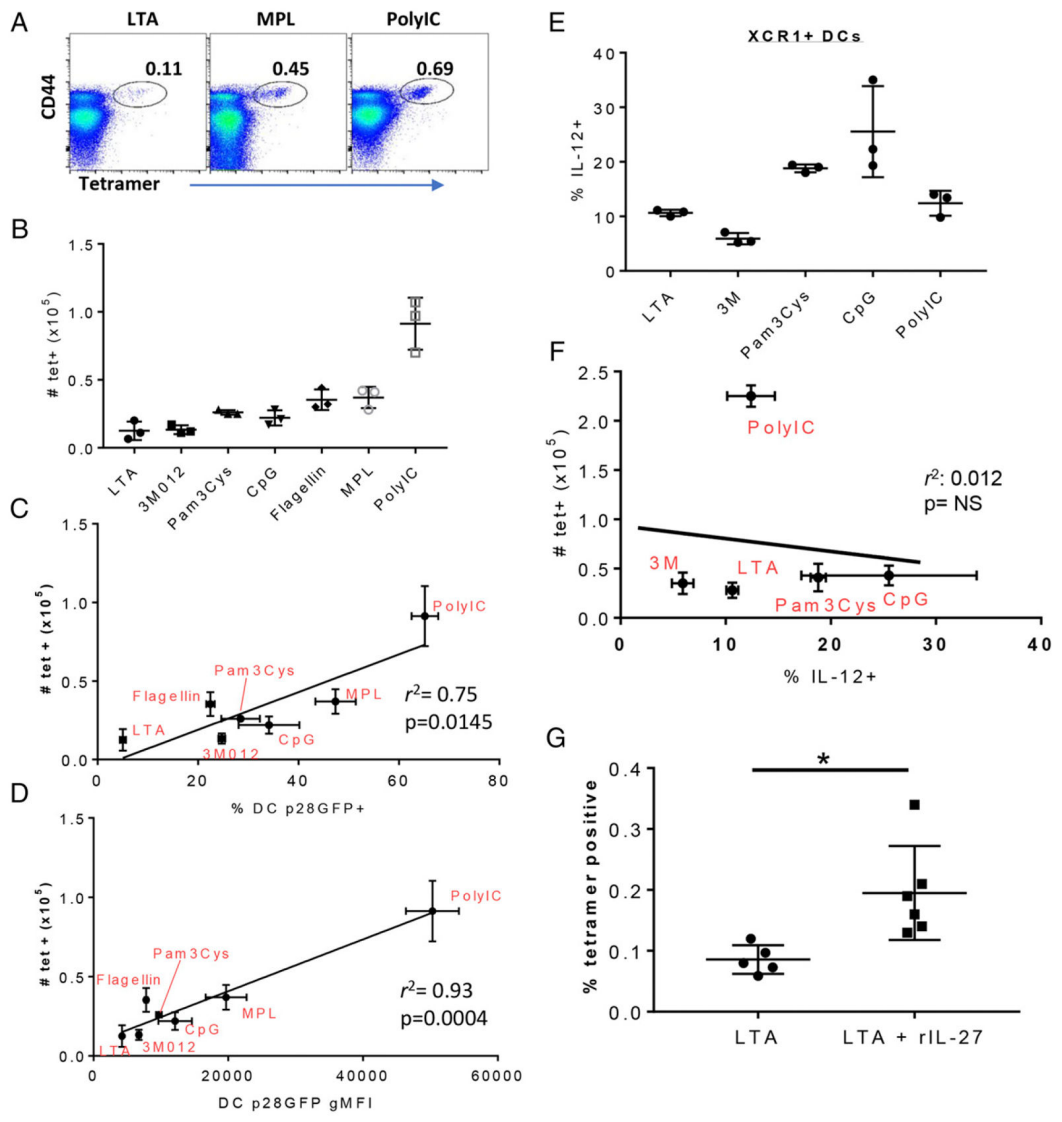
The magnitude of IL-27 produced by cDC1 cells predicts the magnitude of the vaccine adjuvant-elicited CD8^+^ T cell response Mice were immunized i.v. with OVA plus the indicated adjuvants. Seven days later, the spleens were harvested and stained with SIINFEKL–MHC class I K^b^ tetramers, as previously described (20), to identify OVA-specific T cells. (**A**) Representative dot plots showing tetramer staining on all live singlet B220^−^CD8^+^CD3^+^ spleen cells isolated from mice immunized with the indicated adjuvants. (**B**) Quantification of T cell data shown in (A) after immunization with the indicated adjuvants. Error bars indicate SD derived from three mice each. (**C** and **D**) The number of tetramer^+^ T cells generated by each adjuvant, as shown in (B), was plotted against eGFP gMFI of cDC1 cells (C) or the percentage of eGFP^+^ cDC1 cells (D) 6 h postimmunization with each adjuvant, as shown in Fig. 3. *r*^2^ and *p* values were determined by linear regression analysis (Prism). (**E**) Mice were challenged with the indicated innate stimuli, and the splenocytes were isolated at 6 h, as in Fig. 3. Spleens were incubated in vitro for 6 h in the presence of brefeldin A, after which they were stained to identify XCR1^+/−^ DCs, as in Fig. 2. The cells were then fixed, permeabilized, and stained for intracellular IL-12. The percentage of IL-12^+^ XCR1^+^ DCs is shown. (**F**) Tetramer^+^ T cell numbers in the spleen 7 d after immunization with the indicated adjuvants were plotted against the percentage of IL-12^+^ cells shown in (E). *r*^2^ and *p* values were determined by linear regression analysis (Prism). Data in (E) and (F) are from one of two experiments performed. (**G**) Mice were immunized with OVA + LTA, as in (A), with or without the addition of 10 μg of rIL-27. T cell responses were measured as in (A) and (B). *p < 0.05.

TLR agonists are relatively poor inducers of CD8^+^ T cell responses when used as vaccine adjuvants codelivered with soluble protein Ags, generating Ag-specific responses that are exponentially lower than those induced by infectious agents, such as lymphocytic choriomeningitis virus or *L. monocytogenes* (20, 34, 38). However, these adjuvants do induce detectable responses, with each adjuvant generating a different magnitude of T cell immunity (20). Given the fact that the CD4 and CD8 T cell responses to each of these adjuvants is IL-27 dependent (20), the data above suggest the possibility that an adjuvant’s capacity to induce CD8^+^ T cell expansion might be predicted by its induction of IL-27/eGFP. To test this prediction, we immunized mice with the same adjuvants in the context of the model Ag OVA, an Ag ideally suited to the question at hand, given the extreme sensitivity with which OVA-specific CD8^+^ T cell responses can be detected using class I Kb MHC tetrameric reagents (33). Spleen cells were harvested 7 d after immunization, and the CD8^+^ T cell response was measured by MHC tetramer staining (Fig. 4A). The ensuing CD8^+^ T cell response varied considerably among the adjuvants, from a few thousand (LTA) to ~100,000 (polyIC) (Fig. 4A, 4B). Furthermore, the magnitude of the T cell response to each adjuvant tracked well with the capacity of the adjuvant to induce eGFP expression, achieving a robust correlation coefficient that was highly statistically significant (Fig. 4C, 4D). This *r*^2^ value and its significance was highest when plotting the T cell response against eGFP gMFI in DCs (Fig. 4C) or monocytes (Supplemental Fig. 3B, 3C). In sharp contrast, we observed no correlation with the T cell response when analyzing DC cytokines more conventionally associated with robust CD8^+^ T cell immunity, such as IL-12. Analyzing spleen DC IL-12 production at 6 h after immunization by ICCS (Supplemental Fig. 4A), we noted that XCR1^+^ DCs produced varying amounts of IL-12 in response to administration of the various adjuvants (Fig. 4E). However, the amount of IL-12 produced to each adjuvant failed to correlate in any way to the resulting T cell response 7 d later (Fig. 4F). This was true when analyzing IL-12 production from XCR1^+^ DCs (Fig. 4E, 4F) and XCR1^−^ DCs (Supplemental Fig. 4B) or when analyzing IL-12 gMFI in XCR1^+^ DCs (Supplemental Fig. 4C). Collectively, these data are consistent with the conclusion that IL-27p28 production by DCs and monocytes early after vaccine adjuvant administration is highly predictive of the magnitude of CD8^+^ T cell immunity induced.

Given the strength of this correlation between IL-27p28 and the T cell response, we evaluated whether the addition of rIL-27 might improve the T cell response to an adjuvant, such as LTA, that showed poor IL-27 induction. Mice were immunized with OVA + LTA, with or without the addition of 10 μg of rIL-27 (Fig. 4G). As before, LTA was a poor inducer of a CD8^+^ T cell response, generating a T cell response in <0.1% of total CD8^+^ T cells. However, the addition of rIL-27 significantly improved the response (Fig. 4G). Thus, IL-27p28–eGFP expression is predictive of vaccine adjuvant–elicited CD8^+^ T cell responses, and the amount of IL-27 present can directly influence the magnitude of cellular immunity following vaccine adjuvant administration. Ultimately, these data indicate that induction of IL-27 expression in cDC1 cells and monocytes is a reasonable proxy for the capacity of innate receptor-targeting adjuvants to induce cellular immunity.

## DISCUSSION

Our previous study showed the central importance of IL-27 for facilitating vaccine-elicited T cell responses. This was an unexpected finding, given the substantial number of reports on the role of IL-27 as an inhibitor of cellular responses (18, 19, 39–48). Thus, in the absence of IL-27 or its receptor, T cell responses to a host of infectious challenges tended to be elevated, not reduced. Precisely why an adjuvanted subunit vaccine diverges so dramatically from the infectious response with regard to its dependency on IL-27 is still under investigation. Regardless, it serves to highlight the fact that T cell responses to infectious challenge are controlled by different mechanisms than those produced in response to vaccine adjuvants (49). The data that we present in this article show that IL-27 is central to the induction of cellular immunity to subunit vaccination, as well as highly predictive of the resulting overall magnitude of immunity achieved.

Innate immune correlates of vaccine-induced cellular responses have been frustratingly elusive for those invested in the study and implementation of vaccine adjuvants. An excellent example can be found in the innate and adaptive responses produced following administration of an imidazoquinoline TLR7 agonist (50–55), such as the 3M012 used in this study. In only 2–8 h after TLR7 agonist administration, type I IFN, IL-12, and TNF-α are strongly induced from plasmacytoid DCs and cDC2 cells (50, 51). Concomitant with this burst of cytokine production, cDC2 cells are induced to migrate into the T cell regions of the local secondary lymphoid tissues (50), and cDC1 and cDC2 cells increase the expression of molecules important in the costimulation of T cells, such as CD80/86, CD40, and classes I and II MHC (50, 51). Thus, TLR7 stimulation in vivo makes professional APCs migrate into the T cell regions of lymphoid tissues, all while producing soluble and membrane-associated molecules critical for T cell interaction and stimulation. However, as we (33, 53–55) and other investigators (52) have shown, this degree of innate activation is not matched by an equally impressive adaptive response, typically producing T cell responses substantially <1% of the total CD8^+^ T cell pool (Fig. 4). This magnitude of the response is not improved, even when using peptide-based Ags that do not require cross-presentation (33). Similar innate and adaptive results can be obtained for the majority of other innate receptor-based adjuvants (20, 34), consistent with the broad conclusion that the degree of innate immunity generated from vaccine adjuvants, as conventionally measured, is neither commensurate with nor predictive of the ensuing adaptive cellular response. To our knowledge, our data are the first to identify a cytokine, IL-27p28, as an effective and reproducible innate correlate of the adaptive cellular response.

The broad practical conclusion to be drawn from these data is that IL-27 production could be used to screen for novel adjuvants with the capacity to induce T cell immunity. Identifying this correlation is timely, given the growing importance of immunotherapies that rely on augmentation of T cell function for therapeutic efficacy (56, 57). Although highly successful, the efficacy of checkpoint blockade therapies in metastatic cancers is by no means assured (58). The concomitant use of vaccination to further enhance T cell expansion/function will undoubtedly be of great use in this clinical setting, requiring one to question how to select the best adjuvants for just such a purpose. Recent discoveries have focused on identifying mutated tumor Ags and the epitopes within them that might serve as T cell targets (59–62). In the wake of the technical tour deforce that it takes to identify and select these Ags and epitopes, less attention has been paid to the vaccine methods by which the T cell responses against these Ags will be raised. This is despite the fact that the vaccine adjuvant and/or its formulation can dictate the success or failure of the T cell response and resulting therapeutic efficacy. Indeed, a good example of this is an elegant study by Overwijk and colleagues (63), which showed that the robust CD8^+^ T cell response to a cancer-specific epitope could be completely abolished simply by formulating the combination adjuvant in IFA instead of in PBS. This change in formulation effectively created an Ag depot that attracted and induced apoptosis in the T cells generated by the vaccine. It is worth emphasizing that the epitope used in those studies, much like the OVA used in this study, is highly immunogenic. We (64) and other investigators (65) have found similar results using IFA, highlighting the reality that the response to even the best of T cell epitopes can be compromised through the use of the wrong adjuvant. The knowledge that IL-27p28 is a clear indicator of an adjuvant’s capacity to promote T cell responses should be highly useful in the efforts to fully leverage novel Ags and epitopes for maximal clinical benefit.

The fact that adding rIL-27 to LTA improved the ensuing CD8^+^ T cell response indicates at least some degree of causation, not just correlation, between the amount of IL-27 induced by the adjuvant and the cellular response generated. That said, the addition of rIL-27 does not equally improve the T cell response to all adjuvants, with the results generally following a law of diminishing returns for those adjuvants that induce high levels of IL-27 on their own. This is perhaps not unexpected, given the number of other factors known to influence a T cell response (e.g., CD80/86, CD27, efficiency of cross-presentation), making all the more surprising the predictive strength of IL-27p28 across such a broad range of adjuvants.

Finally, it is important to note that this IL-27:CD8 T cell response correlation was generated using all soluble vaccine adjuvant and Ag formulations. We expect that this correlation can be positively and negatively affected depending on other factors, formulation (as highlighted above), or the use of additional coactivators. It remains to be determined whether the use of combined adjuvants might produce T cell responses that deviate from the correlation with the amount of IL-27 that they induce. In our experience, combining innate receptor-specific adjuvants typically produces only additive effects on the magnitude of the T cell response (34). Thus, it seems likely that the T cell correlation with IL-27 production will be reasonably robust across a spectrum of adjuvants and adjuvant combinations, a prediction that is currently under investigation.

## Supplementary Material



## Abbreviations used in this article

B6: C57BL/6
BAC: bacterial artificial chromosome
DC: dendritic cell
gMFI: geometric mean fluorescence intensity
ICCS: intracellular cytokine staining
LTA: lipoteichoic acid
polyIC: polyinosinic-polycytidylic acid
qRT-PCR: quantitative real-time PCR
WT: wild-type
