# Supplementary material for: IL-27p28 Production by XCR1+ Dendritic Cells and Monocytes Effectively Predicts Adjuvant-Elicited CD8+ T Cell Responses
Source: Immunohorizons. Author manuscript; Available in PMC 2018 Jan 17. (PMC5771264; doi:10.4049/immunohorizons.1700054)
Supplement: Supplementary file 1 [file NIHMS931604-supplement-supplement_1.pdf]

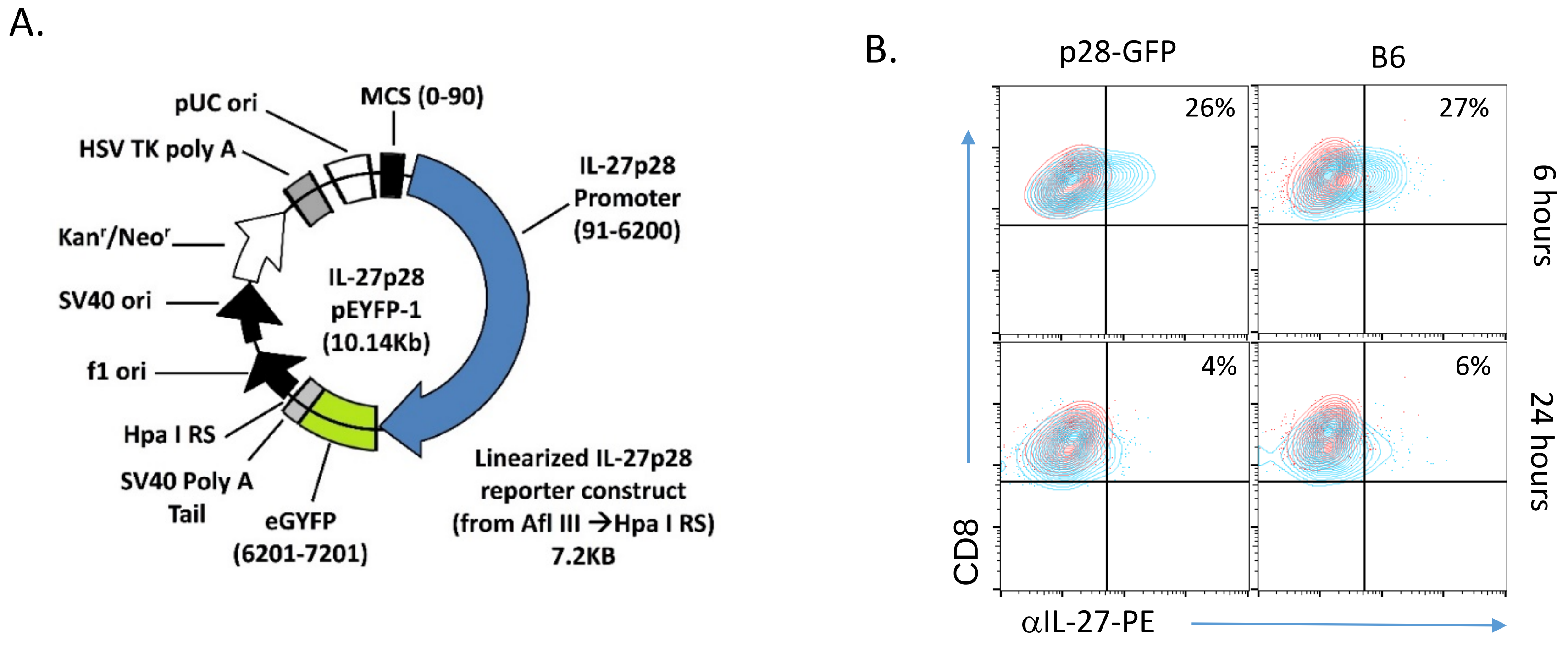

Supplemental Figure 1. **A**. Schematic of the BAC construct designed to express GFP under control of the IL-27p28 promoter region. **B**. IL-27 is not regulated differently in p28GFP mice comparted to WT B6. WT B6 and p28-GFP mice were immunized with polyIC/αCD40 as described in Figure 1 and either 6 or 24 hours later the spleens harvested, collagenase digested and incubated in vitro for 6 hours in the presence of brefeldin A. The cells were then stained with the markers shown in Figure 2A to identify XCR1+ DCs, fixed, permeabilized, and stained with an antibody against p28 (Biolegend). The p28 antibody fluorescence in the DCs is shown from unimmunized (red contours) and immunized (blue contours) p28-GFP (left column) or WT B6 (right column) mice

A.

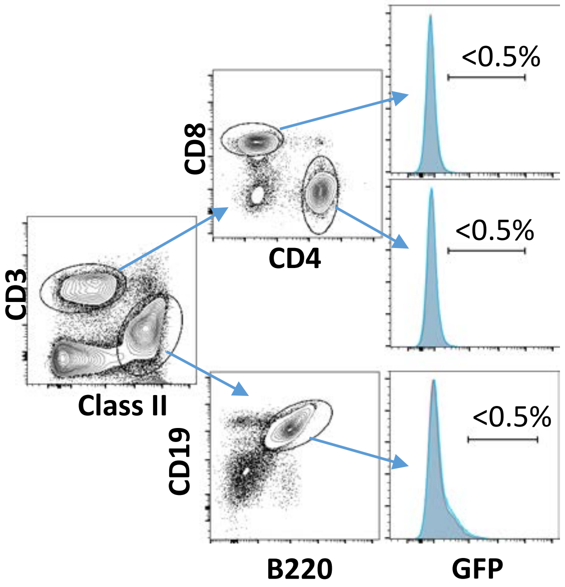

B.

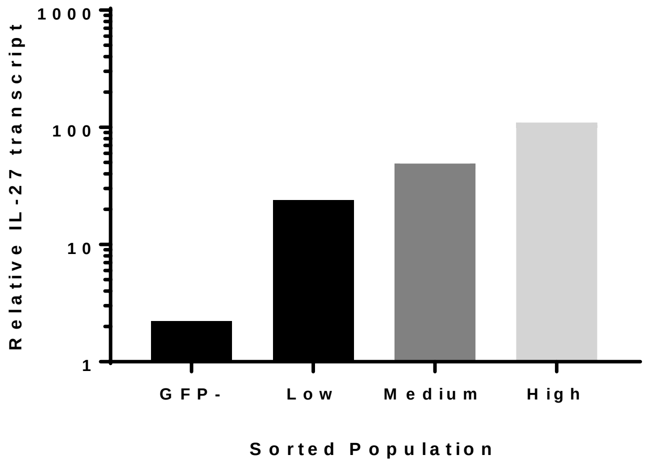

Supplemental Figure 2. A. Lymphocytes (CD4, CD8 or B cells) do not express significant IL-27 based on the GFP reporter. Spleen cells from the immunized mice in Figure 2 were stained with the markers shown to identify T cells and B cells. The GFP expression in the CD4, CD8 and B cells (gated as shown) was compared between unimmunized (red histograms) and immunized (blue histograms) reporter mice. The histogram marker was set based on the unimmunized sample and the number represents the percent GFP+ in the immunized sample. **B.** Levels of GFP expression predict levels of IL-27p28 message. Mice were immunized with polyIC and 6 hours later splenocytes were isolated, flow sorted for different levels of GFP, and the RNA isolated as described in Figure 1. qRT-PCR for p28 was performed as described in the Materials and Methods.

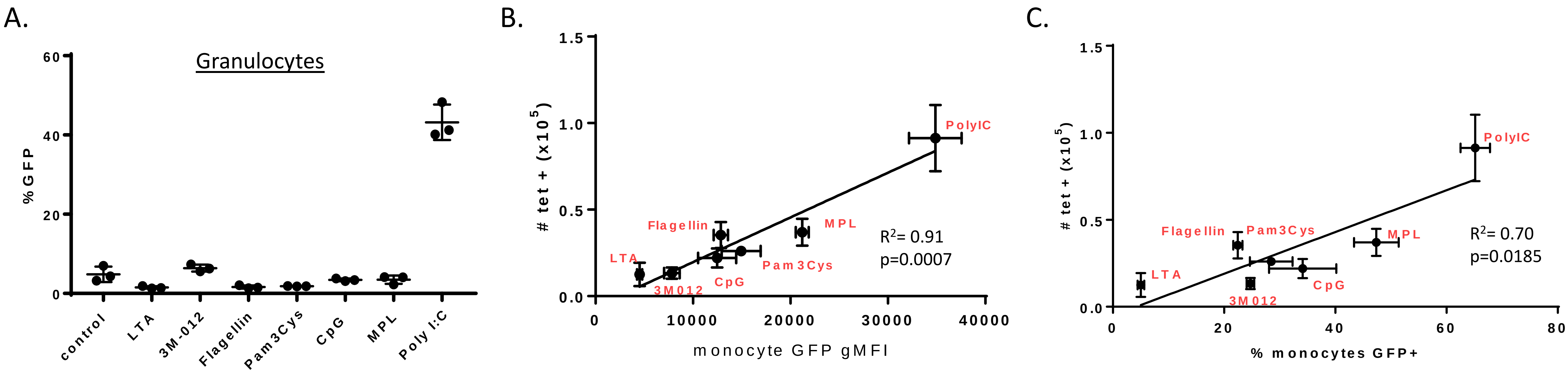

Supplemental Figure 3. A. Of the adjuvants tested, only PolyIC induces IL-27 in granulocytes. Mice were immunized IV with the indicated adjuvants and the spleens harvested 6 hours later. Splenocytes were isolated by collagenase digestion and stained to identify granulocytes as described in Figure 2. Granulocytes were evaluated for both %GFP+ by flow cytometry. The experiment was performed twice, error bars indicating standard deviation derived from 3 mice each. **B and C**. The magnitude of IL-27 produced by monocytes predicts the magnitude of the vaccine adjuvant-elicited CD8+ T cell response**.** The number of tetramer + T cells generated by each adjuvant, as shown in Figure 4, was plotted against either GFP gMFI (B) or %GFP+ (C) of the monocytes 6 hours post immunization with each adjuvant as shown in Figure 3. r^2 and p value were calculated by linear regression analysis (Prism

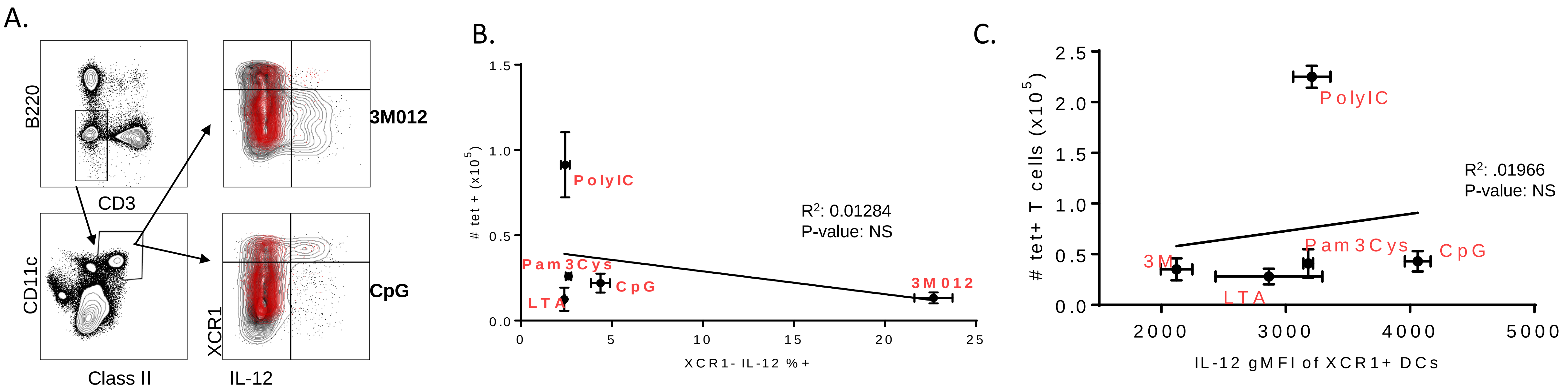

Supplemental Figure 4. IL-12 production from DCs does not predict the CD8+ T cell response to vaccination. **A and B.** Mice were challenged with the indicated innate stimuli and the splenocytes isolated at 6 hours as in B. Spleens were incubated in vitro for 6 hours in the presence of brefeldin A after which they were stained to identify XCR1+/- DCs as in Figure 2,. The cells were then fixed, permeabilized and stained for intracellular IL-12. Representative contour plots from mice immunized with 3M012 or CpG are shown in D overlayed with DCs from unimmunized control spleens in red. **B.** graph of the T cell numbers in the spleen 7 days after the immunization with the indicated adjuvants against the percent IL-12+ in the XCR1- DCs from the experiment shown in Figure 4E. **C.** graph of the T cell numbers in the spleen 7 days after the immunization with the indicated adjuvants against the IL-12 gMFI in the XCR1+ DCs from the experiment shown in Figure 4E.
